# Hot Air Convective Drying of Ginger Slices: Drying Behaviour, Quality Characteristics, Optimisation of Parameters, and Volatile Fingerprints Analysis

**DOI:** 10.3390/foods12061283

**Published:** 2023-03-17

**Authors:** Ruoxi Bai, Jieru Sun, Xuguang Qiao, Zhenjia Zheng, Meng Li, Bin Zhang

**Affiliations:** 1 Key Laboratory of Food Processing Technology and Quality Control in Shandong Province, College of Food Science and Engineering, Shandong Agricultural University, No. 61, Daizong Road, Tai’an 271018, China; 2 Key Laboratory for Applied Technology of Sophisticated Analytical Instruments, Shandong Analysis and Test Center, Qilu University of Technology (Shandong Academy of Sciences), Jinan 250014, China

**Keywords:** ginger, LF-NMR, quality properties, response surface methodology, GC-IMS

## Abstract

Ginger is one of the most popular spices and medical herbs with its unique pungent flavour and taste. Although there has been much research into the drying methods of ginger, the effect of drying parameters in hot air convective drying on ginger quality needs to be explored in depth. This study investigated the differences in drying behaviour and quality characteristics of ginger with the variables of temperature, thickness, and loading density. The moisture states and diffusion pattern in the different stages during the drying process were analysed using low-field NMR techniques. The results of quality evaluation showed that the temperature greatly influenced the colour and gingerol content of dried ginger, and the thickness of a ginger slice greatly influenced the rehydration rate. Optimal drying conditions were determined by considering a combination of specific energy consumptions with quality retention based on the response surface methodology: a temperature of 66.41 °C, thickness of 2 mm, and loading density of 5 kg/m^2^. HS-GC-IMS combined with multivariate chemometrics was used to achieve the characterisation of flavour profiles and fingerprinting of dried ginger. The principal component analysis and correlation analysis revealed that the alterations in ginger quality were intimately related to moisture diffusion during drying.

## 1. Introduction

Ginger, the rhizome of *Zingiber officinale* (Zingiberaceae), is widely used as a seasoning or spice for food and as an herb for medicine [1]. In recent years, it has received extensive attention due to its anti-inflammatory, antipyretic, immunoregulatory, antitumor, antioxidant, hypoglycaemic, and antibacterial properties [2]. The nutritional value of ginger is derived from its complex bioactive components, primarily including gingerols, shogaols, paradols, and zingerone, and its flavour sources are mainly sesquiterpenes and monoterpenes [3]. It is important to note that these components are susceptible to change during storage and processing. Therefore, it is necessary to deeply explore the measures and optimisation strategies to maintain the bioactive compounds of ginger.

The high moisture content (85–95% wet basis, wb) in fresh ginger rhizome makes it susceptible to microbial spoilage and chemical deterioration [4]. Drying (which can remove 90% of water from food) is a crucial way to control the moisture content and extend the shelf life of ginger [5]. With drying, the desirable product quality was protected such as the colour, flavour, nutrients, and texture. In addition, drying reduces the bulk volume and weight of the sample, which is beneficial for packaging and transportation [6,7,8]. Currently, sun drying is a widespread conventional method of ginger drying for many developing countries due to low investment and simple operation. However, its unsecured sanitary environment and uncontrollable drying conditions also limit the quality advancement of ginger. Hot air convective drying (HACD) is a technique of dehydration by diffusion of moisture within the sample through the transfer of heat [9]. It is the most adopted technique in dry product processing plants because of its fast heat transfer and stable temperature. However, it causes an unfavourable thermal degradation of product quality. Therefore, it is essential to choose the proper drying conditions in order to avoid the degradation in flavour, colour, and nutritional components and increase in energy consumption. Low-field nuclear magnetic resonance (LF-NMR) technology has great potential for characterising moisture changes during food drying due to its characteristics of fast speed, high sensitivity, sample retainment, and low cost. It has been successfully used for the real-time detection of water mobility and distribution during the drying of carrot, banana, Pleurotus eryngii, shiitake mushroom, garlic, and burdock [10,11,12]. Moreover, the selection of the optimal drying parameters not only focuses on the independent variables, but also their possible interactions. The response surface methodology (RSM) is considered a widely used, effective statistical method for optimising complex processes [13]. It can describe the comprehensive effects of multiple variables and the interrelationships between variables through a rational experimental design [14]. Due to its advantages of high efficiency, low cost, and convenient experimentation and interpretation, RSM has been applied in chemical component extraction and processing technology optimisation [15,16]. To the best of our knowledge, the combination of the above two practical tools has not been used to study the ginger drying process.

Aroma is one of the most sensitive indicators to judge the quality of various foods and condiments, and special changes will occur during the drying process [17]. Therefore, the identification of volatile components (VOCs) in food is conducive to accurately describing the relationship between the changes in VOCs and food quality. In a past report, Pang et al. [18] identified components affecting the aroma of ginger using gas chromatography—olfactometry (GC-O). Johnson et al. [19] identified 100 volatile components in dried Australian ginger by gas chromatography-mass spectrometry (GC-MS). Yu et al. [20] used headspace gas chromatography-mass spectrometry (HS-GC-MS) and fast GC e-nose to distinguish the varieties and geographical origin of ginger. The GC-IMS method, which is an emerging detection technique, can be used to achieve rapid, real-time identification of volatile compounds based on differences in ion mobility rates under ambient pressure in weak electric fields and in combination with gas chromatography [21]. The advantages of GC-IMS for its simplicity, sensitivity, and rapidity are widely used in food origin labelling [22], freshness evaluation [23], food authenticity identification [24], and monitoring of changes in volatile compounds during processing [25]. Li et al. [26] successfully established a fingerprint of volatile components in the cured ginger process by GC-IMS and interpreted the influence of the curing process on the flavour characteristics of ginger. Nevertheless, the application of GC-IMS in ginger drying is less reported and correlation analysis with drying and quality parameters has not been reported.

The aim of this work was to explore the optimisation and evaluation of the drying process of ginger and to analyse the differences in volatile compounds between fresh and dried ginger. The effects of three variables (drying temperature, thickness, and load density) on the drying rate and quality (colour, brown index value, rehydration rate, and gingerol content) of ginger were evaluated. The transverse relaxation time (T2) combined with the PLSR (partial least squares regression) model was used to analyse the moisture status and water loss pattern during the drying process. In addition, the optimal drying conditions were obtained by considering a combination of specific energy consumptions with total gingerol content based on RSM. Then, HS-GC-IMS combined with multivariate chemometrics was used to achieve the characterisation of flavour profiles and fingerprinting of ginger.

## 2. Materials and Methods

### 2.1. Sample Preparation

Fresh ginger was purchased from a local supermarket in Laiwu (Shandong Province, China) and stored in a refrigerator at 4 °C for no more than seven days before drying. In the drying experiments, ginger pieces of the same size (the diameter was about 3.0 cm) were washed and cut into 2, 4, and 6 mm slices. They contained 91% moisture (wet basis, wb) by drying at 105 °C in a hot air convective drying oven (Yiheng Technology Co., Ltd., Shanghai, China) to constant weight.

### 2.2. Drying Experiments

Fresh ginger slices were dried in a laboratory-scale HACD (Yiheng Technology Co., Ltd., Shanghai, China) at an air velocity of 0.8 m/s (Figure S1). According to preliminary tests and previous studies, the drying temperatures were set at 60, 70, and 80 °C and the loading densities were set at 3, 4, and 5 kg/m^2^, respectively [27]. In the drying chamber, the relative humidity of the environment was maintained at 15 to 20%. The samples were weighed every 0.5 h under different drying conditions until the final moisture level reached below 10% (wb). Each experiment was performed in triplicate.

### 2.3. Drying Curves

Moisture content (*MC*) (dry basis, db), moisture ratio (*MR*), and drying rate (*DR*) were calculated using Equations (1)–(3), respectively [28].

(1) $$\mathrm{MC}=\frac{W_{t}-W_{d}}{W_{d}}$$

(2) $$\mathrm{MR}=\frac{M_{t}-M_{e}}{M_{0}-M_{e}}$$

where *MC* and *MR* are the moisture content (g/g, db) and the moisture ratio (%), respectively; *W_t_* (g) and *W_d_* (g) are the mass at any time and the constant mass after drying at 105 °C, respectively; *M_t_* (g/g, db), *M*_0_ (g/g, db), and *M_e_* (g/g, db) are the *MC* at any time, initially, and at equilibrium, respectively [29].

(3) $$\mathrm{DR}=\frac{M_{t+\mathrm{dt}}-M_{t}}{\mathrm{dt}}$$

where *DR* is the drying rate (g/g min, db); *M_t_*, *M_t+dt_*, and t are the moisture contents (g/g, db) at *t* and *t + dt* and the drying time (h), respectively.

### 2.4. Water Status

An LF-NMR instrument (MesoMR23-060H-I, Niumag Corp., Shanghai, China) was adopted to monitor the water status of the drying process. For T2 determination, the Carr–Purcell–Meiboom–Gill (CPMG) pulse sequence was used, with the typical parameters of TW = 2500 ms, TE = 0.2 ms, NECH = 14,000, and NS = 8.

### 2.5. Colour

The colours of the ginger slices were measured using a NH310 high-quality portable colorimeter (Shenzhen 3NH Technology Co. Ltd., Shenzhen, China). A D65 light source was used, with a measuring aperture of φ8 mm. Before measuring, a white standard plate was used to calibrate the chroma meter. The CIE LAB colour parameters L* (whiteness), a* (redness), and b* (yellowness) were used to express the colours of the samples. The total colour difference (∆*E*) and browning index (*BI*) were calculated by Equations (4)–(6) to describe the colour changes [30]:

(4) $$\Delta E=\sqrt{{\left(L^{*}-L_{0}^{*}\right)}^{2}+{\left(a^{*}-a_{0}^{*}\right)}^{2}+{\left(b^{*}-b_{0}^{*}\right)}^{2}}$$

where the subscript 0 refers to the values of the parameters of fresh ginger.

(5) $$\mathrm{BI}=\frac{100(x-0.31)}{0.17}$$

(6) $$x=\frac{a^{*}+1.75L^{*}}{5.645L^{*}+a^{*}-3.012b^{*}}$$

### 2.6. Rehydration Coefficient

The rehydration coefficient *(RC*) was used to determine the rehydration capacities of dried ginger slices. The dried ginger slices were placed in 250 mL of 95 °C constant-temperature water for 10 min. After removal from the water, the surface moisture was removed by blotting with filter paper, and the rehydrated samples were weighed. Each experiment was performed in triplicate. The *RC* values were calculated using Equation (7) [5]:

(7) $$\mathrm{RC}=\frac{W-W_{0}}{W_{0}}$$

where *W (g)* and *W*_0_ (g) are the weight of the rehydrated sample and the initial weight of the dried sample, respectively.

### 2.7. Specific Energy Consumption

Specific energy consumption (*E_kg_*) is the energy consumption necessary for drying 1 kg of raw ginger. To determine the precise power consumption, the electricity meters (DDS7738, Shanghai Li Hua Electric Meter Factory, Shanghai, China) were attached directly to the drying oven. *E_kg_* was calculated using Equation (8) [31]:

(8) $$E_{\mathrm{kg}}=\frac{P\times t}{W_{0}}$$

where *P* is the power (kW), *t* is the drying time (h), and *W*_0_ is the initial weight of the sample (kg).

### 2.8. Gingerol Contents

Ginger samples (0.5 ± 0.01 g) were mixed with 75% methanol (25 mL) in a conical flask with a cap. Then, the samples were ultrasonically extracted (KQ-500DE ultrasonic bath, Kunshan Ultrasonic Instrument Co. Ltd., Shanghai, China) at 50 °C and 200 W for 40 min. Furthermore, after centrifugation at 5500× *g* for 10 min, the filtrate was filtered through a 0.22 μm organic membrane filter to obtain the extract. High-performance liquid chromatography (HPLC) (Shimadzu Corp., Kyoto, Japan) was used to determine the contents of 6-gingerol, 8-gingerol, and 10-gingerol in the ginger extracts. A C_18_ column (Phenomenex Corp., Torrance, CA, USA; 4.6 mm × 250 mm, i.d., 5 μm) was used for chromatographic separation. The mobile phase was prepared using acetonitrile (A) and water (B). The gradient elution programme was as follows: 0–30 min, 35–70% A; 30–40 min, 70% A; 40–45 min, 70–100% A. The flow rate was set at 1 mL/min, the injection volume was 20 μL, and the column temperature was maintained at 30 °C. The peaks for gingerol content were monitored at 280 nm.

### 2.9. Box–Behnken Design (BBD)

A Box–Behnken design (BBD) with three factors (*X*_1_, drying temperature (°C); *X*_2_, thickness (mm); *X*_3_, loading density (kg/m^2^)) at three levels was performed to optimise the conditions of drying ginger in HACD. As shown in Table 1, two dependent variables (specific energy consumption and gingerol content) were taken as the response for acquiring a statistical model. A total of 15 experimental tests (including 3 centre points) are presented in each response surface analysis using a full quadratic equation as follows [32].

(9) $${Y=b}_{0}+\sum_{j=1}^{k}b_{j}X_{j}+\sum_{j=1}^{k}b_{\mathrm{jj}}X_{j}^{2}+\sum \;\sum_{i<j}b_{\mathrm{ij}}X_{i}X_{j}$$

where *Y*, *X_i_*, and *X_j_* represent the response variables, and the independent variables *b*_0_*, b_j_, b_jj_*, and *b_ij_* are the constant coefficients.

### 2.10. GC-IMS Analysis Parameters

A 0.5 g (d.b.) sample of ginger in the fresh and dry state were placed in a 20 mL headspace vial and incubated at 80 °C for 10 min, respectively. After incubation, the 300 μL headspace extractions were injected into an injector automatically by a syringe with a temperature of 85 °C and separated by a GC column (FS-SE-54-CB-0.5, 15 m × 0.53 mm × 0.5 μm). The carrier gas was high-purity N2 (99.999%) with the flow at 2 mL/min for 2 min, then increasing to 100 mL/min for 23 min, and finally being maintained at 150 mL/min for 10 min. The total GC runtime was 35 min. The pre-separated ions were driven to the 9.8 cm drift tube after a 3H ionisation source (500 V) in positive ion mode with a temperature of 45 °C and the nitrogen flow rate was 150 mL/min. The VOCs were identified by the RIs of standard substances in the GC-IMS library (G.A.S.) and the drift time of experimentally determined measures.

### 2.11. Statistical Analysis

The results are expressed as the mean ± standard deviation (SD) and were subjected to analysis of variance (ANOVA) tests in triplicate. Differences were considered significant at the 95% confidence level (*p* < 0.05). Identification of volatile compounds and the establishment of fingerprint profiles were carried out using GC-IMS library search software and LAV software (G.A.S., Dortmund, Germany).

## 3. Results

### 3.1. Drying Characteristics

**Figure 1:**
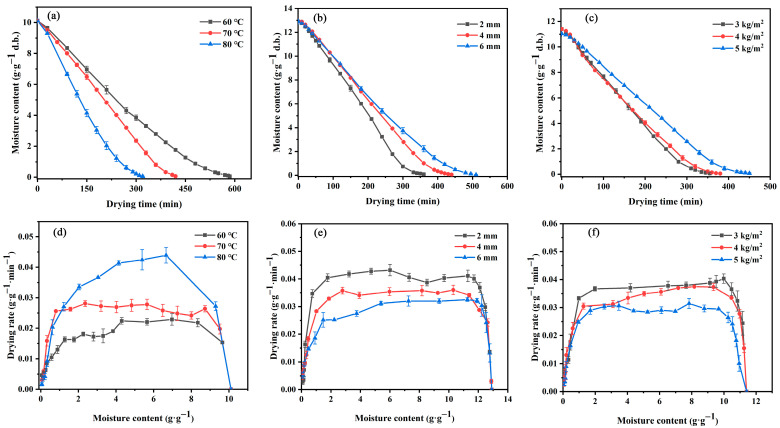
(**a**–**c**) Drying curves and (**d**–**f**) drying rate curves of ginger at different temperatures, thicknesses, and loading densities.

In this study, three drying variables were selected to evaluate the drying characteristics of ginger during the HACD drying process: temperature (60, 70, and 80 °C), thickness (2, 4, and 6 mm), and loading density (3, 4, and 5 kg/m^2^). As shown in Figure 1a–f, the drying curves and drying rate curves of ginger revealed that the moisture content under the three variables gradually decreased with the extension of drying time. Compared with that at 60 °C, the drying times at 70 and 80 °C were reduced by 28.2% and 58.8%, respectively. In contrast, the effects of thickness and loading density on the drying time were positively correlated. The shortest drying times were observed when the thickness was 2 mm and the loading density was 3 kg/m^2^. Furthermore, the drying rate increased with increasing drying temperature, while it had negative correlations with the thickness and the loading density. It is worth noting that a constant-rate phase followed by a rate-reducing phase was found during the drying process, which is consistent with the findings of Mahayothee et al. [27]. It suggests that the mechanism of the removal of water is internal diffusion. It was also found that the higher temperature led to a shorter duration of the constant-velocity phase, but the thickness and loading had limited effects on the drying rate. Therefore, temperature is the main factor affecting the drying of ginger in hot air, and there were two stages in the drying process: surface vaporisation control and internal diffusion control.

### 3.2. Evaluation and Prediction of Water State by LF-NMR

**Figure 2:**
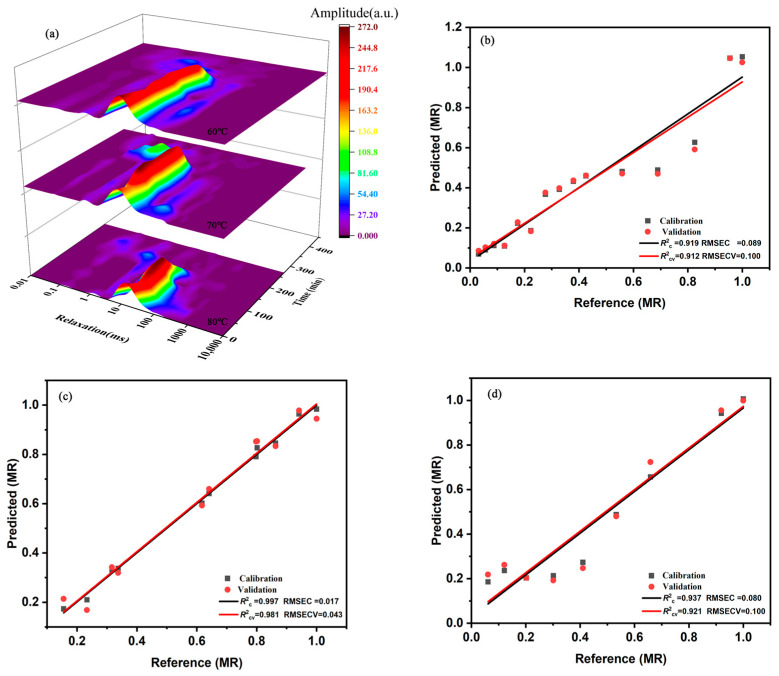
(**a**) Transverse relaxation time (T2) 3D colour map for ginger dried at different temperatures. Relationship between measured values and *MR* values predicted using the PLSR models: (**b**) 60 °C, (**c**) 70 °C, and (**d**) 80 °C.

The water state and distribution during the drying process followed a basic mechanism [33]. Figure 2a show a clear display of T2 on the waterfall colour map during the HACD drying process. There were three different water states during the ginger drying process: T21 in the range of 0.01–1 ms indicated a bound state of water (bound water), T22 at approximately 10 ms indicated an immobile state of water (immobile water), and T23 at approximately 100 ms indicated a free state of water (free water). Their ratio in fresh ginger slices was 0.72:12.79:86.49 (T21:T22:T23, %), indicating that the water in ginger was mainly free water. According to the freedom degree (T2) of water and the moisture content (A2, area of each peak) at different temperatures, the mobility of water increased due to heating in the early stages of drying. This result is in agreement with the transient rise in the drying rate shown in Figure 1d–f. In addition, relatively balanced moisture diffusion stages were found at all three temperatures during the drying process, where only free water (A23) was slowly reduced. This phase ended at 270, 180, and 90 min for the drying temperatures of 60, 70, and 80 °C, respectively. Notably, the corresponding drying rate also had a relative equilibrium period at the uniform drying stage. This indicated that the constant rate of ginger drying is dominated by the reduction in free water on the sample surface, which is consistent with the drying rate curve. After reaching a critical point, A23 dropped rapidly, and A22 and A21 also dropped slowly. This was the decelerated drying phase, and the internal diffusion of the material was the dominant factor [34].

Although LF-NMR could be used to analyse and characterise the drying behaviour of ginger, it was necessary to develop a supervised quantitative method to predict the moisture contents of unknown samples. A correction model for predicting the moisture content of ginger was established using PLSR based on the strong collinear properties of NMR signal parameters [35]. According to the NMR calibration model for *MR* at 60, 70, and 80 °C (Figure 2b–d), the *R*^2^c values were 0.919, 0.997, and 0.937; the *R*^2^cv values were 0.912, 0.981, and 0.921; the RMSEC values were 0.089, 0.017, and 0.080; the RMSECV values were 0.100, 0.043, and 0.100, respectively. The higher *R*^2^ and lower RMSE values indicated that the PLSR model exhibited good performance with regard to calibration and cross-validation. Therefore, LF-NMR was an effective method for analysing and predicting the HACD drying process of ginger.

### 3.3. Colour and BI

Colour is an important attribute that determines the acceptability of the sample [9]. As shown in Table 2, the colour parameters and browning index of dried ginger under the three variables changed greatly after drying. Under different conditions, ΔL*, Δa*, and Δb* values ranged from –9.30 ± 0.03, 1.63 ± 0.03, and –1.50 ± 0.19 to –4.99 ± 0.07, 3.14 ± 0.05, and –0.36 ± 0.18. Therefore, the dried sample showed a darker, redder, and bluer colour than the fresh sample did. The significantly higher variation degree of ΔL* than those of Δa* and Δb* indicated that the brightness played a major role in the colour change during drying. Temperature had a large effect on colour change, and the maximum value of Δ*E* occurred at 80 °C. Moreover, the temperature and load variables significantly affected the *BI* value (*p* < 0.05). The dried ginger processed at 80 °C had the maximum *BI* value (49.91 ± 0.50). As reported by Martins et al. [36], the changes in the colour parameters and *BI* values at high temperatures can be mainly attributed to the nonenzymatic browning reactions of food.

### 3.4. Rehydration Coefficients

Rehydration determinations were conducted to evaluate the qualities of the dried ginger slices obtained under different drying conditions. Table 2 shows the results of the rehydration coefficients of dried ginger under the three variable conditions of temperature, thickness, and loading. Overall, both the maximum and minimum rehydration coefficients (5.57 ± 0.16 and 3.34 ± 0.36, respectively) were found in the thickness variable group, with a significant difference (*p* < 0.05). In contrast, no significant differences were detected in the rehydration rates under the temperature variable (*p* > 0.05), and differences produced by loading were not always significant. This suggested that slice thickness was the main factor affecting the rehydration rate of food products, and they exhibited a negative correlation. This phenomenon might be attributed to the structural modifications in the food during drying that affect the rehydration capacity [5]. For large-thickness samples, a longer drying time resulted in sealed surface capillaries [37]. In contrast, severe volume shrinkage and compact internal structures appeared in small-thickness dried samples, which had enhanced water absorption due to the pumping function created by rehydration. Therefore, it is necessary to select an appropriate slice thickness during the drying process.

### 3.5. Gingerol Content

6-Gingerol, 8-gingerol, and 10-gingerol are the main phenolic substances in ginger, and they are also relatively abundant in dried ginger [38]. As shown in Table 2, there was no significant difference in the contents of the three gingerols at different loading densities (*p* > 0.05). Under different thickness conditions, the contents of 6-gingerol, 8-gingerol, and 10-gingerol were 8.25 ± 0.11–9.08 ± 0.38 mg/g, 2.05 ± 0.01–2.96 ± 0.90 mg/g, and 4.24 ± 0.18–4.89 ± 4.65 mg/g, respectively. Only the 10-gingerol contents under the thickness variables of 2 and 6 showed a significant difference with that under the thickness variable of 4 (*p* < 0.05). Therefore, the loading density and the thickness had relatively small effects on the overall content of gingerol. Furthermore, a positive correlation between the overall gingerol content and the temperature was observed. When ginger was dried at 60 °C, the overall gingerol content of the obtained product was smallest, with a 6-gingerol content of 5.78 ± 0.24 mg/g, 8-gingerol content of 1.27 ± 0.11 mg/g, and 10-gingerol content of 2.48 ± 0.25 mg/g. The reason for this might be that the longer drying time caused a greater decomposition of gingerol. The maximum 6-gingerol content (7.97 ± 0.63 mg/g) of dried ginger processed at 70 °C is consistent with the results of previous findings [4]. Compared with the 6-gingerol content, the contents of 8-gingerol and 10-gingerol were relatively stable. In summary, temperature was the main factor affecting the overall gingerol content in dried ginger.

### 3.6. Optimisation of Drying Conditions by RSM

#### 3.6.1. Specific Energy Consumption

**Figure 3:**
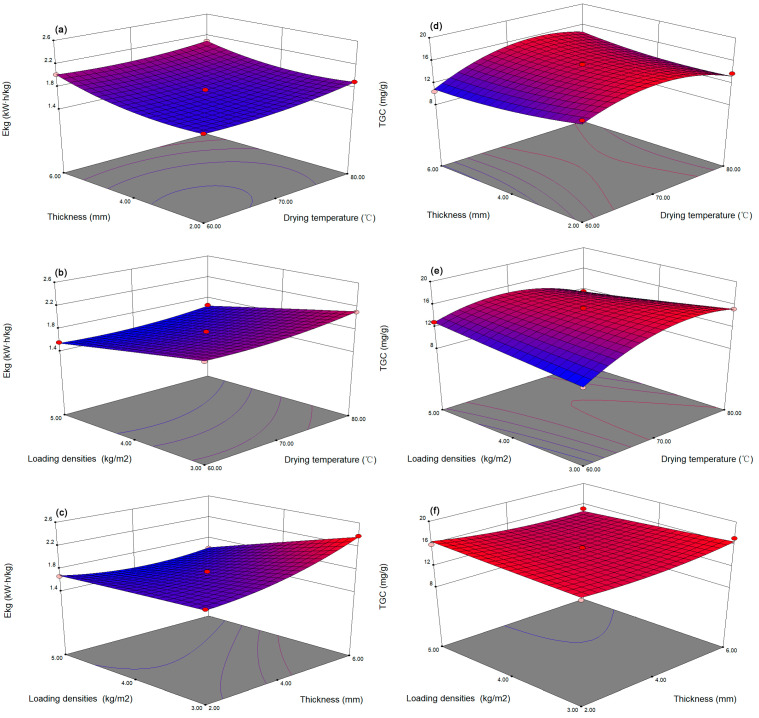
Response surface plots of operating parameters (**a**–**c**) for *E_kg_* and (**d**–**f**) for TGC.

Drying is a highly energy-intensive process, so energy consumption is a necessary indicator for determining whether a drying method is reasonable [39]. Figure 3a–c show the effects of drying temperature, thickness, and loading density on the *E_kg_* of dried ginger. *E_kg_* was positively correlated with temperature. When the temperature increased from 60 to 80 °C, *E_kg_* increased significantly by 11.66% (Table 3). Furthermore, *E_kg_* was lower than 1.7 kW h/kg for ginger with thicknesses of 2–3 mm, and a further increase in thickness caused *E_kg_* to be as high as 2.15 kW h/kg. *E_kg_* was reduced by 20% with respect to the loading density as the load increased from 3 kg/m^2^ to 5 kg/m^2^. The results of the ANOVA for the *E_kg_* of the dried samples obtained by HACD drying are given in Table S1. In the analysis, only the variable *X*_1_*X*_3_ was not significant (*p* > 0.05). This was due to the increase in temperature that shortened the drying time and ultimately decreased *E_kg_*. The higher R^2^ of *E_kg_* (0.9981) and the nonsignificant lack-of-fit term (0.0718) confirmed that the model fit well and was appropriate for the experimental data. According to the F value representing the effects of the variables on the response values, the variables corresponded to *E_kg_* in the order of *X*_3_ > *X*_2_ > *X*_1_. A *p* value less than 0.05 indicated that the model term was significant, and a *p* value less than 0.01 showed that the model term was extremely significant. After the application of the response surface regression process, the predicted polynomial equation was as shown:

(10) $$\begin{array}{c} E_{\mathrm{kg}}=1.74+0.069X_{1}+0.13X_{2}-0.22X_{3}-0.031X_{1}X_{2}-0.021X_{1}X_{3}-0.16X_{2}X_{3}\\ +0.064X_{1}^{2}+0.11X_{2}^{2}-3.292\times 10^{-3}X_{3}^{2} \end{array}$$

#### 3.6.2. Total Gingerol Content

The total gingerol content (TGC) was determined by summing each of the three gingerol contents. Figure 3d–f and Table 3 suggest that the temperature exerted a significant effect on the TGC (*p* < 0.05). As the temperature increased from 60 to 80 °C, the TGC showed a trend of first increasing and then decreasing. The maximum value (21.37 mg/g) of dried ginger obtained at 70 °C might be attributed to the highest content of 6-gingerol. Compared with that of the dried ginger with a thickness of 2 mm, the TGC of the dried ginger with a thickness of 6 mm was reduced by 44.04% because the longer drying time necessary for the increased slice thickness was detrimental to the stability of gingerol. In contrast, the loading density had little effect on the TGC. In the interaction effect, only the effect of *X*_1_*X*_3_ on the TGC was significant (*p* < 0.05). Table S2 shows the ANOVA results for the TGCs in the dried samples obtained by HACD drying. The R^2^ value was 0.9501, and the lack of fit was not significant (*p* > 0.05). *X*_1_ had the largest F value, indicating the significant effect of the temperature on the TGC. The full quadratic model was obtained as:

(11) $$\;\mathrm{TGC}=15.05+0.93X_{1}-0.25X_{2}-0.27X_{3}+0.93X_{1}X_{2}-1.65X_{1}X_{3}-0.68X_{2}X_{3}\;-2.75X_{1}^{2}+0.69X_{2}^{2}+0.049X_{3}^{2}$$

#### 3.6.3. Determination and Experimental Validation of Optimal Conditions

To meet the requirements of energy savings and quality assurance in actual production, the minimum *E_kg_* and maximum TGC values were used to optimise the drying conditions of the samples. The optimal drying conditions obtained by RSM were as follows: a temperature of 66.41 °C, thickness of 2 mm, and loading density of 5 kg/m^2^, with an *E_kg_* and a TGC of 1.65 kW h/kg and 16.68 mg/g, respectively. Comparatively, the final experimental values were 1.78 ± 0.04 kW h/kg and 18.54 ± 1.18 mg/g, respectively. They were essentially consistent with no significant differences within a 95% confidence interval, indicating the applicability of RSM in optimising the conditions of the HACD drying process of ginger.

### 3.7. The Volatile Compounds Identified and the Volatile Fingerprints under Fresh and Dried Ginger by HS-GC–MS

**Figure 4:**
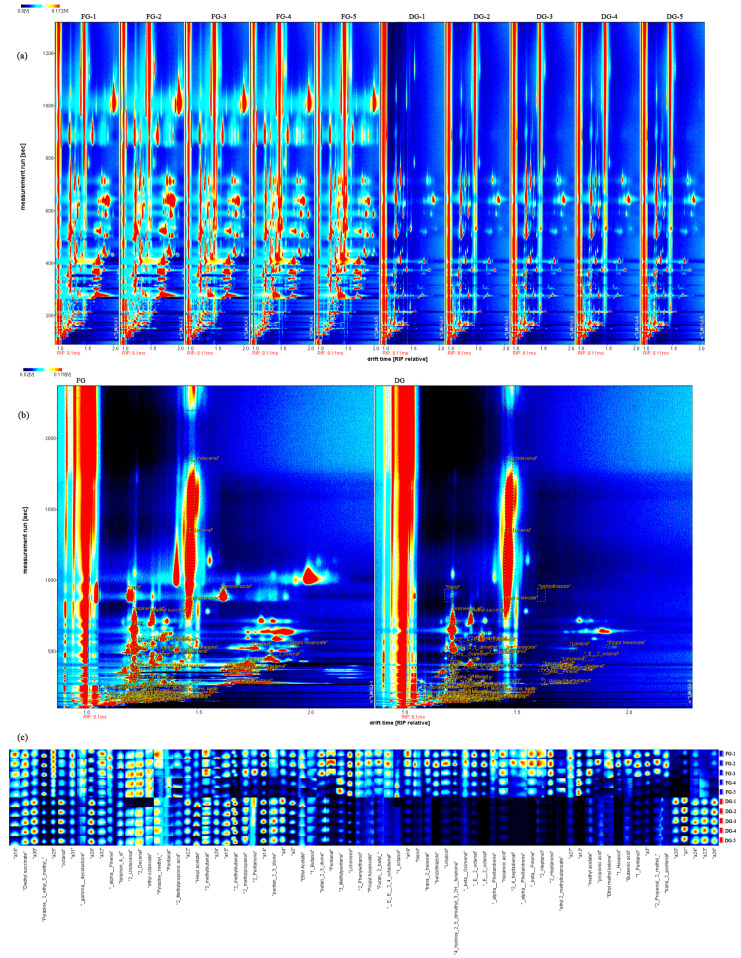
(**a**) Topographic plots of volatile compounds in fresh and dried ginger; (**b**) Topographic plots of GC–IMS spectra with the selected markers obtained; (**c**) Fingerprinting plot of VOCs in fresh and dried ginger.

The VOCs of fresh (FG) and dried (DG) ginger were evaluated by HS-GC-IMS. The topographic plots of the VOCs were obtained after normalising the ion drift time and the position of the reaction ion peaks. In the plots, the X-axis indicates the relative drift time and the Y-axis indicates the retention time [40]. Each dot on the diagram represents a volatile component (Figure 4b). The colour of the dots indicates the signal intensity, with blue meaning low intensity and red meaning high intensity. As shown in Figure 4a, the difference in volatile compounds between fresh and dried ginger can be clearly captured. The signals of some VOCs in dried ginger disappeared or were diminished, which is opposite to the VOCs in the fresh samples. The characterisation of VOCs of ginger was obtained by comparing retention index, retention time, and drift time, while the National Institute of Standard and Technology database (NIST) was also utilised. The results of the analysis are shown in Table 4; the fresh and dried ginger contained a wide range of compounds, with 48 different kinds of VOCs identified and 21 peaks not identified. The VOCs of ginger included aldehydes (11), esters (9), alcohols (7), terpenoids (7), ketones (7), acids (3), and others (4), which is consistent with a previous study of ginger in Yu et al. [20]. Further, in order to provide a comprehensive overview of the spots identified in fresh and dried ginger, the fingerprint was established (Figure 4c). In the diagram, one sample was represented with a row and one VOC was represented with a column, and five samples each of fresh and dried ginger were used for evaluation. A noticeable trend was that the concentration of 12 compounds vanished after drying, which were benzothiazole, (E)-2-octenal, α-Phellandrene, hexanoic acid, 2,4-heptadienal, β-Pinene, 2-Heptanone, ethyl 2-methylbutanoate, 1-Hexanol, Nerol, Linalool, 4-hydroxy-2,5-dimethyl-3(2H)-furanone, and 2-Heptanol. In addition, the contents of butan-2,3-dione, pentanal, 3-methylpentane, limonene, 2-phenylethanol, propyl hexanoate, furan, 2-butyl, (2E,4E)-2,4-octadienal, trans-2-hexenal, β-ocimene, methyl acetate, and 1-octanol were dramatically reduced after drying. Nevertheless, the contents of diethyl succinate, hexyl acetate, pyrazine, methyl, 2-methylbutanal, and pentan-2,3-dione were also higher in dried compared to fresh ginger. The above results may be caused by the deoxygenation of compounds produced at high temperatures or the conversion of sesquiterpenoids into monoterpenoids.

### 3.8. Distinction of Fresh and Dried Ginger by PCA Analysis

**Figure 5:**
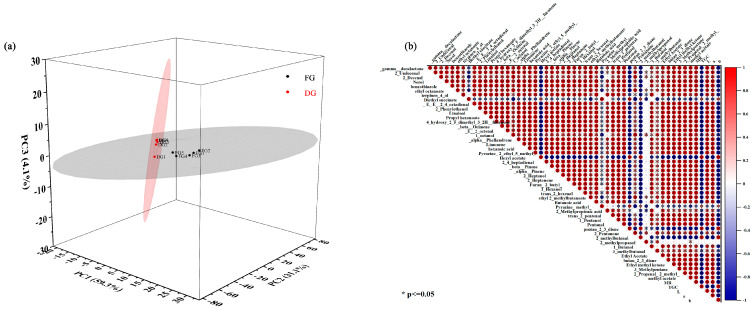
(**a**) PCA plot of VOCs intensity detected by HS-GC-IMS. (**b**) Correlation map of MR, VOCs, and quality parameters according to their Pearson correlation coefficient.

As a well-known stoichiometric dimensionality reduction method, the principal component analysis (PCA) method was used to visualise the large amount of data obtained from HS-GC-IMS results [40]. The results are shown in Figure 5a, and the data basis for the PCA was the peak intensity of each compound in Table 4. The PC1, PC2, and PC3 explained 59.3%, 31.1%, and 4.1% of the accumulative variance contribution rate, respectively. The PC1 and PC2 accounted for 90.4% of the total variance and they were considered adequate for further discussion. As shown in Figure 5a, the five samples were located in close proximity and the fresh ginger and dried ginger were well separated. The results showed that the aroma characteristics of fresh and dried ginger samples were significantly different, and the GC-IMS technique as a sensitive flavour detection tool could achieve rapid determination of VOCs of the samples before and after processing.

### 3.9. Correlation Analysis of Moisture and Quality Indicators Related to Drying

A correlation matrix was used to determine the correlation between moisture and quality parameters of the ginger drying process by the Pearson correlation test. The correlation heat-map in Figure 5b shows that the *MR* was negatively related to the TGC (r = 0.95) and positively related to colour parameters L and a (r = 0.99, and 0.89), but negatively related to b (r = 0.86). In addition, all VOCs in ginger were significantly correlated (*p* < 0.05) with *MR* except for 2-methylpropanol. Among them, diethyl succinate (r = 0.31), hexyl acetate (r = 0.99), pyrazine, methyl (r = 0.24), pentan-2,3-dione (r = 0.84), and 2-methylbutanal (r = 0.99) were negative related to the *MR*, while the other VOCs were positive related to the *MR* (0.41 < r < 0.99). This is consistent with the results of Figure 4c, indicating that the drying treatment increased the content of these VOCs. These correlations found in this study may indicate that the alterations of ginger quality were intimately related to moisture diffusion during drying. Furthermore, GC-IMS data have the potential to effectively distinguish ginger samples with different dryness levels.

## 4. Conclusions

This study integrated several quality attributes to evaluate the three drying variables and optimised the optimal drying process for ginger, and further analysed the differences in volatile compounds of ginger before and after drying. At different variables, the ginger drying process could be divided into three stages: a short-term increasing-rate drying period, a constant-rate drying period, and a rapidly decreasing-rate drying period, where the constant-rate phase is mainly a slow decrease of free water. The optimal drying process was determined by combining multivariate analysis methods: a temperature of 66.41 °C, thickness of 2 mm, and loading density of 5 kg/m^2^. HS-GC-IMS analysis revealed significant differences in volatile compounds between fresh and dried ginger, with heat treatment leading to a decrease in the levels of most terpenoids and aldehydes. In addition, the correlation analysis yielded a close correlation between the moisture content and the changes in volatile compounds. The method established in this study can be further used for the optimisation of other drying techniques for food products and the exploration of the effect of moisture content on key aroma substances.

## Figures and Tables

**Table 1:** Experimental design matrix of HACD drying of ginger.

Variables	Coded Levels		
	−1	0	1
Drying temperature (*X*_1_), °C	60	70	80
Thickness (*X*_2_), mm	2	4	6
Loading density (*X*_3_), kg/m^2^	3	4	5

**Table 2:** Quality parameters of ginger dried at different temperatures, thicknesses, and loading densities.

		L*	a*	b*	Δ*E*	*BI*	*RC*	Gingerol Contents (mg/g)		
								6-Gingerol	8-Gingerol	10-Gingerol
Drying temperature, °C	60	73.43 ± 0.11 ^b^	1.19 ± 0.06 ^b^	28.10 ± 0.18 ^a^	8.36 ± 0.13 ^b^	48.09 ± 0.50 ^b^	4.26 ± 0.09 ^a^	5.78 ± 0.24 ^b^	1.27 ± 0.11 ^c^	2.48 ± 0.25 ^b^
	70	74.50 ± 0.07 ^a^	0.73 ± 0.05 ^c^	28.27 ± 0.01 ^a^	7.22 ± 0.07 ^c^	47.09 ± 0.11 ^c^	4.29 ± 0.06 ^a^	7.97 ± 0.63 ^a^	1.79 ± 0.13 ^b^	3.66 ± 0.25 ^a^
	80	72.10 ± 0.03 ^c^	1.83 ± 0.05 ^a^	28.10 ± 0.20 ^a^	9.83 ± 0.05 ^a^	49.91 ± 0.50 ^a^	4.36 ± 0.17 ^a^	7.51 ± 0.04 ^a^	2.05 ± 0.01 ^a^	3.85 ± 0.01 ^a^
Thickness, mm	2	73.57 ± 0.06 ^b^	0.32 ± 0.03 ^c^	29.24 ± 0.19 ^a^	8.13 ± 0.04 ^a^	49.43 ± 0.40 ^a^	5.57 ± 0.16 ^a^	8.75 ± 0.12 ^ab^	2.96 ± 0.90 ^a^	4.65 ± 0.09 ^a^
	4	73.48 ± 0.19 ^c^	1.09 ± 0.07 ^a^	28.60 ± 0.47 ^b^	8.32 ± 0.22 ^a^	48.98 ± 1.14 ^a^	4.43 ± 0.45 ^b^	9.08 ± 0.38 ^a^	2.38 ± 0.09 ^a^	4.24 ± 0.18 ^b^
	6	75.93 ± 0.03 ^a^	0.81 ± 0.07 ^b^	28.28 ± 0.09 ^b^	5.89 ± 0.04 ^b^	46.07 ± 0.26 ^b^	3.34 ± 0.36 ^c^	8.25 ± 0.11 ^b^	2.55 ± 0.04 ^a^	4.89 ± 0.07 ^a^
Loading densities, kg/m^2^	3	75.97 ± 0.11 ^b^	0.60 ± 0.03 ^a^	29.45 ± 0.20 ^a^	6.00 ± 0.05 ^b^	48.18 ± 0.35 ^a^	4.08 ± 0.03 ^b^	7.06 ± 0.03 ^a^	2.03 ± 0.01 ^a^	3.95 ± 0.02 ^a^
	4	76.41 ± 0.07 ^a^	0.37 ± 0.02 ^c^	28.38 ± 0.10 ^b^	5.30 ± 0.06 ^c^	45.46 ± 0.17 ^b^	4.63 ± 0.03 ^a^	6.95 ± 0.46 ^a^	1.92 ± 0.13 ^a^	3.75 ± 0.24 ^a^
	5	75.21 ± 0.18 ^c^	0.47 ± 0.01 ^b^	26.97 ± 0.19 ^b^	6.49 ± 0.20 ^a^	43.64 ± 0.23 ^c^	4.14 ± 0.04 ^b^	7.23 ± 0.73 ^a^	2.01 ± 0.16 ^a^	3.88 ± 0.26 ^a^

Each value is the mean ± standard deviation (*n* = 3). Different letters in the same column indicate a significant difference (*p* < 0.05).

**Table 3:** Experimental factors and measured values of the responses.

Run	Independent Variables			Responses	
	*X*_1_	*X*_2_	*X*_3_	Ekg (kw·h/kg)	TGC (mg/g)
1	0	−1	1	1.56	21.37
2	−1	−1	0	1.64	17.75
3	1	0	−1	1.92	16.71
4	0	−1	−1	1.72	20.12
5	1	−1	0	1.54	13.07
6	0	0	0	1.73	11.88
7	0	0	0	1.75	10.16
8	0	1	1	1.72	12.59
9	0	0	0	1.77	11.35
10	1	0	1	1.7	15.74
11	1	1	0	1.94	13.69
12	−1	1	0	1.74	10.07
13	0	1	−1	2.15	11.26
14	−1	0	1	1.74	10.33
15	−1	0	−1	1.78	9.23

**Table 4:** The presence of a variety of volatile compounds in fresh and dried ginger, including alcohols, aldehydes, terpenoids, esters, and acids.

NO	Compounds	CAS	Molecule Formula	MW	RI	Rt	Dt
1	γ-decalactone	706-14-9	C_10_H_18_O_2_	170.3	1480.7	2285.21	1.4903
2	2-Undecenal	2463-776	C_11_H_20_O	168.3	1383.1	1578.444	1.4865
3	2-Decenal	3913-71-1	C_10_H_18_O	154.3	1278.8	1063.094	1.4749
4	Nerol	106-25-2	C_10_H_18_O	154.3	1230.9	886.403	1.2046
5	benzothiazole	95-16-9	C_7_H_5_NS	135.2	1230.9	886.403	1.6216
6	ethyl octanoate	106-32-1	C_10_H_20_O_2_	172.3	1208	812.781	1.4672
7	terpinen-4-ol	562-74-3	C_10_H_18_O	154.3	1174.4	715.601	1.2239
8	Diethyl succinate	123-25-1	C_8_H_14_O_4_	174.2	1179.8	730.325	1.3012
9	(2E,4E)-2,4-octadienal	30361-28-5	C_8_H_12_O	124.2	1109.3	559.081	1.2677
10	propyl	60-12-8	C_8_H_10_O	122.2	1107.4	555.006	1.3064
11	Linalool	78-70-6	C_10_H_18_O	154.3	1092.5	524.443	1.7565
12	Propyl hexanoate	626-77-7	C_9_H_18_O_2_	158.2	1098	535.649	1.9235
13	4-hydroxy-2,5-dimethyl-3(2H)-furanone	3658-77-3	C_6_H_8_O_3_	128.1	1078.3	496.936	1.1927
14	β-Ocimene	13877-91-3	C_10_H_16_	136.2	1053.9	453.128	1.2193
15	(E)-2-octenal	2548-87-0	C_8_H_14_O	126.2	1056.3	457.203	1.3355
16	1-octanol	111-87-5	C_8_H_18_O	130.2	1063.8	470.447	1.4661
17	α-Phellandrene	99-83-2	C_10_H_16_	136.2	1012.2	386.907	1.2226
18	Limonene	138-86-3	C_10_H_16_	136.2	1025.1	406.264	1.7326
19	hexanoic acid	142-62-1	C_6_H_12_O_2_	116.2	1019.1	397.095	1.6439
20	Pyrazine, 2-ethyl-5-methyl-	13360-64-0	C_7_H_10_N_2_	122.2	997.2	365.512	1.1734
21	Hexyl acetate	142-92-7	C_8_H_16_O_2_	144.2	1007.3	379.775	1.4148
22	2,4-heptadienal	5910-85-0	C_7_H_10_O	110.2	998	366.531	1.6193
23	β-Pinene	127-91-3	C_10_H_16_	136.2	972.4	340.77	1.6457
24	α-Pinene	80-56-8	C_10_H_16_	136.2	929.2	302.153	1.2181
25	2-Heptanol	543-49-7	C_7_H_16_O	116.2	897.9	276.918	1.7266
26	2-Heptanone	110-43-0	C_7_H_14_O	114.2	887	269.271	1.6372
27	Furan, 2-butyl	4466-24-4	C_8_H_12_O	124.2	888.2	270.036	1.1777
28	1-Hexanol	111-27-3	C_6_H_14_O	102.2	861	253.595	1.333
29	trans-2-hexenal	6728-26-3	C_6_H_10_O	98.1	841.6	242.507	1.182
30	ethyl 2-methylbutanoate	7452-79-1	C_7_H_14_O_2_	130.2	847	245.566	1.2369
31	Butyric acid	107-92-6	C_4_H_8_O_2_	88.1	833.3	237.919	1.164
32	Pyrazine, methyl	109-08-0	C_5_H_6_N_2_	94.1	819.9	230.655	1.0868
33	2-Methylpropionic acid	79-31-2	C_4_H_8_O_2_	88.1	791.7	216.126	1.3677
34	trans-2-pentenal	1576-87-0	C_5_H_8_O	84.1	743.2	194.332	1.1061
35	Pentanol	71-41-0	C_5_H_12_O	88.1	758.3	200.832	1.2583
36	Pentanal	110-62-3	C_5_H_10_O	86.1	704.3	178.596	1.1866
37	pentan-2,3-dione	600-14-6	C_5_H_8_O_2_	100.1	692.1	173.952	1.297
38	Propyl methyl ketone	107-87-9	C_5_H_10_O	86.1	699.2	176.648	1.3769
39	2-methylbutanal	96-17-3	C_5_H_10_O	86.1	645.5	163.468	1.4048
40	2-methylpropanol	78-83-1	C_4_H_10_O	74.1	654.8	165.415	1.1671
41	1-Butanol	71-36-3	C_4_H_10_O	74.1	661.9	166.913	1.1832
42	3-methyl butyraldehyde	590-86-3	C_5_H_10_O	86.1	638.9	162.12	1.1846
43	Ethyl Acetate	141-78-6	C_4_H_8_O_2_	88.1	614.4	157.177	1.0974
44	butan-2,3-dione	431-03-8	C_4_H_6_O_2_	86.1	603	154.93	1.1765
45	Ethyl methyl ketone	78-93-3	C_4_H_8_O	72.1	600.7	154.481	1.253
46	3-Methylpentane	96-14-0	C_6_H_14_	86.2	582.9	151.036	1.2154
47	2-Propenal, 2-methyl-	78-85-3	C_4_H_6_O	70.1	559.7	146.693	1.2167
48	methyl acetate	79-20-9	C_3_H_6_O_2_	74.1	521.7	139.803	1.1957

## Data Availability

The data presented in this study are contained within the article.

## Supplementary Materials

The following supporting information can be downloaded at: https://www.mdpi.com/article/10.3390/foods12061283/s1, Table S1: ANOVA results for *E_kg_* of the dried samples obtained under hot air convective drying; Table S2: ANOVA results for TGC of the dried samples obtained under hot air convective drying; Figure S1: Diagram of hot air convective drying system.
