# Supplementary material for: Hot Air Convective Drying of Ginger Slices: Drying Behaviour, Quality Characteristics, Optimisation of Parameters, and Volatile Fingerprints Analysis
Source: Foods. 2023 Mar 17;12(6):1283. doi: 10.3390/foods12061283 (PMC10047944; doi:10.3390/foods12061283)

Table S1 ANOVA results for E_{kg} of the dried samples obtained under hot air convective drying.

Source	Sum of squares	Df	Mean square	F-value	P-value Prob > f	
Model	0.71	9	0.079	288.73	<0.0001	**
X_1	0.038	1	0.038	139.49	<0.0001	**
X_2	0.13	1	0.13	469.15	<0.0001	**
X_3	0.38	1	0.38	1366.54	<0.0001	**
X_1X_2	0.00391	1	3.91×10^{-3}	14.2	0.013	*
X_1X_3	0.00172	1	1.72×10^{-3}	6.26	0.0543	
X_2X_3	0.11	1	0.11	382.86	<0.0001	**
X_1^2	0.015	1	0.015	55.35	0.0007	**
X_2^2	0.048	1	0.048	175.11	<0.0001	**
X_3^2	4.00×10^{-5}	1	4.00×10^{-5}	0.15	0.7186	
Residual	0.00138	5	2.75×10^{-4}			
Lack of fit	0.00131	3	4.36×10^{-4}	13.08	0.0718	Not significant
Pure error	6.67×10^{-5}	2	3.33×10^{-5}			
Total	0.72	14				
			$R^2 = 0.9981$			

Level of significance *, $P < 0.05$; **, $P < 0.01$.

Table S2 ANOVA results for TGC of the dried samples obtained under hot air convective drying.

Source	Sum of squares	Df	Mean square	F value	P value Prob > f	
Model	53.58	9	5.95	8.67	0.0142	*
X_1	6.92	1	6.92	10.08	0.0247	*
X_2	0.31	1	0.31	0.46	0.5292	
X_3	0.84	1	0.84	1.23	0.3182	
X_1X_2	3.48	1	3.48	5.07	0.0742	
X_1X_3	10.93	1	10.93	15.92	0.0104	*
X_2X_3	1.35	1	1.35	1.96	0.2203	
X_1^2	27	1	27	39.32	0.0015	**
X_2^2	1.51	1	1.51	2.2	0.1977	
X_3^2	4.72×10^{-6}	1	4.72×10^{-6}	6.87×10^{-6}	0.998	
Residual	3.43	5	0.69			
Lack of fit	2.86	3	0.95	3.34	0.239	Not significant
Pure error	0.57	2	0.29			
Total	57.01	14				
			R^2= 0.9501			

Level of significance *, $P < 0.05$; **, $P < 0.01$.

Figure S1 Diagram of hot air convective drying system.

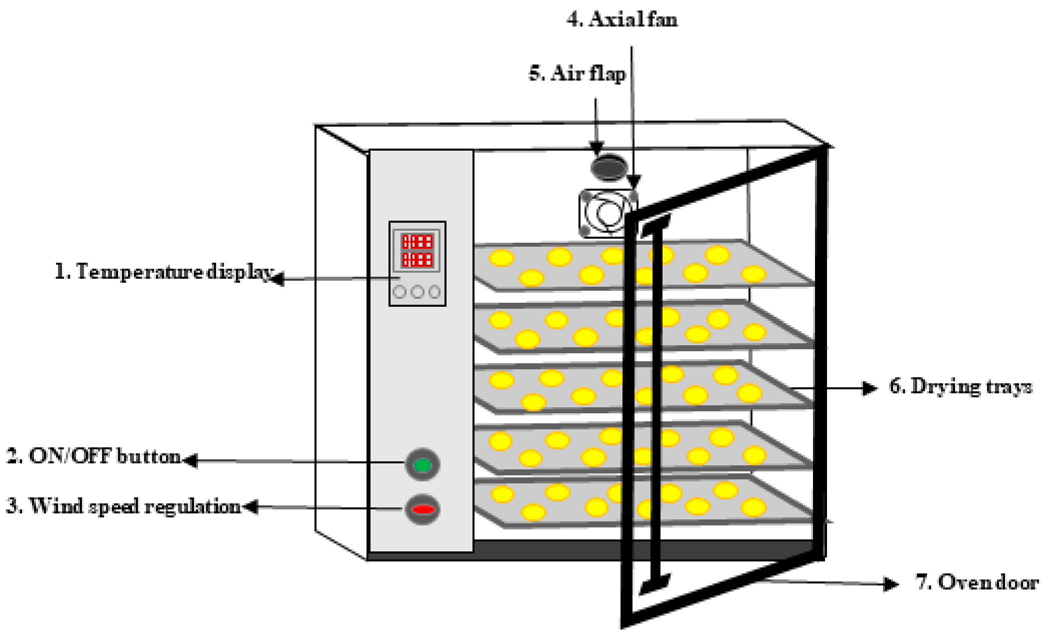

Supplement: Supplementary file 1 [file foods-12-01283-s001.zip › foods-2240760-supplementary.pdf]
